# Medical Items, News and Gossip

**Published:** 1875-08

**Authors:** 


### Medical Items, News and Gossip.

(BY L'ANCIEN EDITEUR.)

It is reported that Dr. Fordyce Barker "produced a profound impression" on the medical mind of London, by a lecture before a learned body in that city, on the treatment of puerperal fever. Whether favorable or unfavorable, the reporter sayeth not. Dr. Barker assumed the "Alonzo Clark doctrine:" that puerperal fever, being essentially of traumatic origin, and its phenomena due to communication to the nervous system of irritation, therefore its remedy is to be sought in opiates to keep the nervous system quiescent until the local injury is remedied by ordinary physiological processes. It is assumed that this treatment has been practiced generally in New York for the last fifteen or twenty years, and therefore excites no surprise in the American mind, although sufficiently disturbant to the Anglican sentiment. The somewhat dubitant Chicagoan marvels not at the "profound impression" of the Anglican mind under the circumstances. —— Transfusion begins to be understood. The late lamented Gen. Blair was temporarily benefited. What more could have been expected? The transfused blood is only an advance on digestible food. In the capillaries even the blood is still *outside* the *living* body. The blood is nothing but nutrient material, ready to be appropriated for nutrition. The "life is in the blood" just as the life of man is in beefsteak, cabbage, and cod-liver oil. Transfusion, to be useful and promising, should be as frequently repeated as the want of the system for food suggests. As far as at present understood, it is comparatively useless, except to make up for the waste of sudden hæmorrhage. Its use in cachexia is simply absurd. —— Bellevue Hospital Medical College sends us an announcement extensive enough for a Common Prayer Book, and promising enough to make all medical neophytes both successful and rich. Fortunately it is FLINT-y at the centre, and therefore may evolve the Promethean spark of prosperity. —— The Woman's Hospital Medical College of this city greets us smilingly for its sixth year. May it succeed! We have always desired for women their appropriate sphere, although differing somewhat from some of our contemporaries as to its real nature. The curriculum seems full and the faculty strong. It is to be regretted that under the heading "Preceptor" there is to be found the name of an infinitesimal specialist of the female persuasion. Will her name be admitted as that of an appropriate "Preceptor" when the student applies for graduation? —— Professors Andrews and Quine, of this city, commend, in special letters, Messrs. Powers & Weightman's Sulphate of Cinchonidia, an article that seems growing in professional favor. —— The establishment of a homœopathic department in the University of Michigan threatens tribulation to our ancient colleagues in the effort to "raise the standard." We extend to the brethren our sympathies. —— The Alabama State Medical Association offers a prize of one hundred dollars, or a gold medal of equal value, for the best essay on "Bright's Disease." Anybody anywhere can compete. —— The recent remarkable appointment of American medical students to positions in the Khedive's Army, in Egypt, proves the fraudulent device of a scheming impostor. There were no "millions in it." —— Prof. Hammond has resigned the editorial charge of the *Journal of Psychological Medicine aud Medical Jurisprudence*, and has been succeeded by Allen McL. Hamilton. —— It is some comfort to learn that jaborandi, the new Brazilian medicine, in doses of fifty grains, will, in fifteen minutes, cause profuse sweating and excessive flow of saliva. "Sixteen ounces of distinctly alkaline saliva were collected in a few hours, and the sweating was so great as to soak the bed clothes." In addition to its moistening qualities, in large doses, jaborandi "causes tension of the accommodative apparatus of the eye." —— The *American Practitioner* (July) contains, from the pen of Lunsford P Yandell, M.D., a highly appreciative Memoir of the Life and Writings of Dr. John Esten Cooke. Medical biography has few names that deservedly rank higher. Three or four decades ago, and his opinions had larger influence in shaping Western and Southern practice than perhaps any other. All the older practitioners will readily recall his favorite pills, calomel, rhubarb and aloes. He thoroughly believed in calomel and the apostolical succession. —— Our Canada *confrere* is out strongly in favor of cremation. It prefers Sieman's furnace to a coffin, and lively caloric to six feet of superimposed soil. Every one to their taste. In the thronged districts of the old world there is abundant argument for incineration, but on this continent there seems room and verge enough to allow poor bodies to rot at leisure. Time enough and space enough for that last banquet, where, as Hamlet observed, one does not eat, but is eaten. —— The *Boston Journal of Chemistry* attributes the late disastrous explosion of the Dows apothecary store, in that city, to the ignition of the vapor of ether with atmospheric air. One part of the former to six or eight of the latter affords a powerful and dangerous explosive agent. Our ether-eal anæsthetic friends will please make a note of this. The argument is not all *versus* chloroform. —— It is rumored that two of the occupants of important chairs in a well-known medical college have tendered their resignations. Known as long-time incumbents, there is much anxiety to ascertain what *is* or *was* in the wind. Before another issue, it is hoped the atmosphere will be calm. —— Another case of entire absence of the pancreas is recorded—this time in Paris. —— The venerable Dr. Bullard, of New Haven, Vt., who has been the earliest acquaintance of over a thousand youngsters, is to be complimented soon by a grand reunion pic-nic. The *Philadelphia Medical Times* suggests that *the* toast of the occasion will be : "The tie that binds us—Dr. Bullard—our common deliverer." —— Prof. Zeissl, of Vienna, is said to believe syphilis absolutely ineradicable from the system that has once received it. At a clinic recently, he exclaimed: "Some think when a patient has for sometime enjoyed immunity from manifestations of syphilis, that he is cured; but I tell you, gentlemen, that if a man contracts syphilis he will die syphilitic, and at the day of judgment his ghost will have syphilis!" It is to be concluded, however, a "spiritual manifestation" thereof. —— Alfred Heap, an abortionist, was hung at Manchester, England, last April, for his crime. —— Analogous to the writer's palsy comes the newly noted disease, "telegraph operator's cramp." —— M. GALLARD (*The Doctor*) uses iodoform crayons, prepared by mixing iodoform in fine powder with gum arabic mucilage, shaping and drying *secund. art.* The crayons are to be kept in dark air-tight bottles, to prevent disintegration. —— The *Danbury News* Man thinks the recovery of a baby from convulsions is an illustration of the Darwinian doctrine of "survival of the *fitist.*" —— Another batch of four babies at one confinement is chronicled by the *Detroit Review.* Daughters — two placentæ; labor, an hour and a half; avoirdupois, twenty-four pounds. —— "There is a soul of goodness in things evil." A recent writer asserts that "the blue mould of cheese (aspergillus glaucus) is considered by epicures to give the cheese an agreeable flavor, and it is probable that it helps the digestion of other aliments. Again, a good portion of the stinking rust in flour is supposed not to render it unwholesome, at least when made into fermented bread. The flour is largely used in the manufacture of gingerbread, where the treacle disguises the color and flavor." Which, also, suggests another probable truth, that living spores, fungi, animalculæ and wigglers, *et id omne genus*, are not generally disease-producing, but evidences of the presence of decomposing, organic matter about always disease producing, but out of which these atoms of life get nourishment and size sufficient to lead ætiologists into funny misapprehensions. —— Dr. DELAFIELD read a paper recently before the

Med. Soc. of the Co. of New York, in which he showed by statistics, that in pneumonia, the largest number of fatal cases occurred on the 7th day, the next largest on the 14th, the next on the 21st, and the next on the 28th. The end of each week, therefore, seems critical, and particularly the end of the first week. Perhaps, after all, "the fathers" were wise in their day and generation. —— Dr. Robert C. Kedzie, of the Michigan State Board of Health, referring to poisonous wall paper, shows that arsenical papers are not limited to those of green color, but that all papers of delicate and "toned" colors may be regarded as suspicious. It gratifies the present writer that his whilome pupil and long time friend, Prof. Kedzie, is distinguishing himself by a great variety of practical researches in the etiology of disease. —— *Medicus*, in the *Druggists' Circular*, recommends for sweating feet, the use of ten or fifteen grains of tannin dusted in the bottom of each clean sock—changes to be made every day, and dusting continued for two weeks for permanent cure. It would have been a good thing to have suggested, also, occasional washing of the pedal extremities. —— The slangwhangers of the secular press have seized upon the case of the late Gen. J. C. Breckenridge, as they did upon that of poor "Jim Fisk," to make use of as an excuse for throwing mud at the profession. It is a cheap exercise of penny-a-line wit. It appears that Gen. Breckenridge had, as the result of an old injury received in the army, a hepatic abscess, which unfortunately opened through the bronchial tubes. Professors Sayre and Gross, as a forlorn hope, attempted to establish a more direct outlet for the pus. The operation proved unsuccessful, as the chances were immense that it would, and the poor man died. But it was an opportunity for the turkey-buzzards, and they have improved it. We have only to say, that when our last hours come, or our friends fear their approach, we ask no better counsel (nor could expect it) than that of the illustrious gentlemen whose names have been in this case made a target for abuse in the case of Gen. Breckenridge. —— Who nose? The Chinese, it is said, inoculate by blowing the pulverized virus into the right nostrils of boys and the left ditto of girls. The result is said in each case to be all right. —— The Anthony case, at Leavenworth, is exciting general interest in the profession. That the other editor, shot about the same time, and in whose brains an ounce bullet of lead still remains without producing particular uneasiness, after the old Cavendish crowbar case, does not surprise, but in Col. Anthony, the lacerated subclavian, with its attendant injuries, aneurism and apparent probable cure, awaken much anxiety to read an authorized professional history of the whole matter. —— Dr. Wetherly, of Alabama, asserts, in the *Atlanta Journal*, that so far from quinia being ecbolic as some believe, it is, on the contrary, one of the best agents for arresting uterine action. —— Hydrate of chloral was recently exhibited in a case of infantile convulsions, by Dr. Bemiss (*N. O. Med. & Sur. Journal*) with really remarkable success. After seventeen distinct convulsions, "the child being black from head to foot, the surface cold, and the pulse and respiration completely suspended," Dr. B. recommended using chloral by enema, the condition of the throat being such as to preclude swallowing. An injection containing 1½ grs. of chloral was administered. Relief was immediate. Some hours afterward, a slight convulsion having recurred, the enema was repeated, and after deep sleep, recovery rapidly took place. —— Dr. Geo. J. Huey, in the same journal, strongly advocates quinine in the treatment of pneumonia. He writes: "When called to a patient with cough, pain in one or both sides, fever, with difficulty in breathing, as the general symptoms, I order a dose of the comp. cath. pills of our dispensatory, apply a blister over the seat of pain, and give a cough mixture composed of syrup ipecac and paregoric or laudanum, with directions to give twelve gr. doses of quinine every four hours till three doses are taken—after operation of the pills."

Second day continue treatment, but three doses ten grains each of quinine. Third day, same. Fourth day, same, except eight grain doses of quinine every four hours, etc. etc. Convalescence usually fairly established on the seventh day. No digitalis, verat. virid. or other arterial sedatives, no bleeding or mercury. Undoubtedly a better practice in malarious districts than the old evacuant and sedative plan. —— Our Southern friends are happier because no "iron clad oath" is required at the great Centennial from any one except the U. S. Commissioners, and over these the Directors of the Centennial have no control. But *when* will "the cruel war be over," and iron clad oaths and all other relics of barbarism disappear? —— Dr. A. D. Sinclair (*Bost. Med. and S. Journal*) prefers the fingers above all other methods of dilating the *os uteri*. They certainly have the advantage of always being on hand.

---

